# Intracranial meningeal melanocytoma: a case report and literature review

**DOI:** 10.1093/jscr/rjae332

**Published:** 2024-05-18

**Authors:** Renuka Chintapalli

**Affiliations:** School of Clinical Medicine, Addenbrooke's Hospital, Hills Rd, Cambridge CB20QQ, United Kingdom

**Keywords:** melanocytes, melanoma, supratentorial neoplasm, meningeal neoplasm

## Abstract

Primary intracranial melanocytoma is an uncommon benign pigmented tumor arising from leptomeningeal melanocytes. Neuroimaging characteristics of central nervous system melanocytoma are distinct from similarly presenting intracranial neoplasms and can aid in diagnosis prior to histopathological examination. In rare cases, there may be more than one lesion present. We report a case of a 19-year-old woman presenting with progressively worsening headaches, nausea, emesis, and generalized weakness of 2 months. Imaging revealed tumors in the parietal and ipsilateral medial temporal lobe. The patient underwent gross total resection of the parietal lesion which histopathological assessment revealed to be primary intracranial meningeal melanocytoma. This case highlights the utility of specific imaging criteria such as diffusely increased T1 signal without enhancement in the initial diagnostic evaluation of intracranial melanocytoma. We also describe the clinical characteristics, management strategy, and histopathological features of a rare case of a patient with multiple primary intracranial melanocytoma lesions.

## Introduction

Primary intracranial melanocytoma is a rare type of benign tumor usually found in the posterior fossa, although occurrences in other intracranial compartments have been described [1–3]. These tumors are thought to arise from neural crest-derived melanocytes which are located in the leptomeninges scattered in sulci around the base of the brain and in the upper cervical spinal cord [4]. To date, there are only a few cases of patients with multiple lesions reported in the literature. Here, we report the clinical features, imaging findings, and histopathological characteristics of a patient with primary intracranial meningeal melanocytoma arising in the parietal and temporal lobes.

## Case report

**Figure 1:**
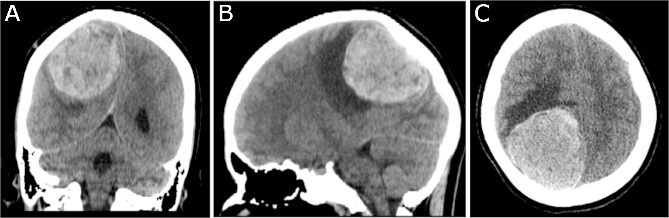
Preoperative CT imaging; (A) coronal, (B) sagittal, and (C) axial CT scans show a large right parietooccipital lesion with vasogenic oedema, mass effect, and midline shift.

**Figure 2:**
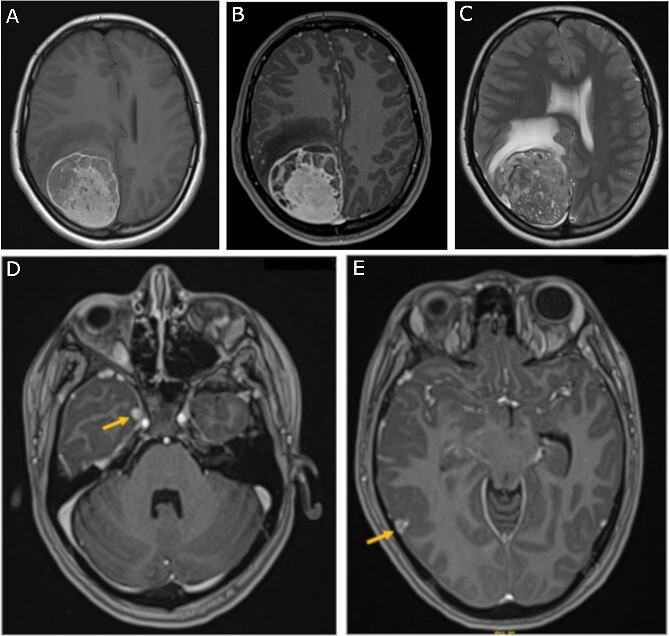
Preoperative MRI; (A) pre-contrast, (B) post-contrast T1-weighted, and (C) T2-weighted axial MRI scans show a large right parietooccipital extra-axial mass with diffusely increased T1 signal without corresponding enhancement; (D, E) axial post-contrast T1-weighted MRI scans show two discrete lobular lesions along the medial aspect of the right temporal lobe and posterior cortical surface of the temporal lobe respectfully.

A 19-year-old woman presented with 2 months of intractable holocephalic headaches associated with nausea, occasional emesis, and generalized weakness which worsened over the 5 days preceding presentation. She had no past medical history and was not taking any regular prescribed medications. A computed tomography (CT) head (Fig. 1) revealed a right parietal mass measuring 6.2 × 5.5 × 5.8 cm. Her magnetic resonance imaging (MRI) re-demonstrated a large right parietal extra-axial mass as well as similar lesions in the right temporal lobe, with diffuse increased T1 signal without corresponding enhancement (Fig. 2).

**Figure 3:**
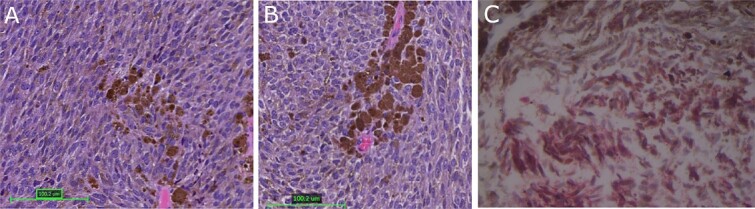
Tumor histopathology and immunohistochemical analysis; (A, B) hematoxylin and eosin-stained sections of heavily pigmented cellular neoplasm with spindled to oval tumor cells with limited cytologic atypia and mitotic count under 0.5 mitoses/mm^2^; (C) immunohistochemistry was positive for Melan A; red color represents MelanA immunopositivity; magnification: 3A: 100×, 3B and 3C: 200×.

The patient underwent a right parieto-occipital craniotomy and gross total resection of the mass. Histological sections (Fig. 3A and B) showed a heavily pigmented cellular neoplasm with spindled to oval tumor cells with limited cytologic atypia and mitotic count under 0.5 mitoses/mm^2^, distinguishing it from malignant melanoma. There was no evidence of brain invasion. Immunohistochemistry was positive for Melan A (Fig. 3C) and HMB45 (data not shown). Cellular proliferation was assessed by staining for Ki-67, which was positive in <2% of tumor cells. Solid tumor next-generation sequencing identified the pathogenic GNAQ mutation pQ209P. The histologic, immunohistochemical, and molecular findings were consistent with meningeal melanocytoma.

**Figure 4:**
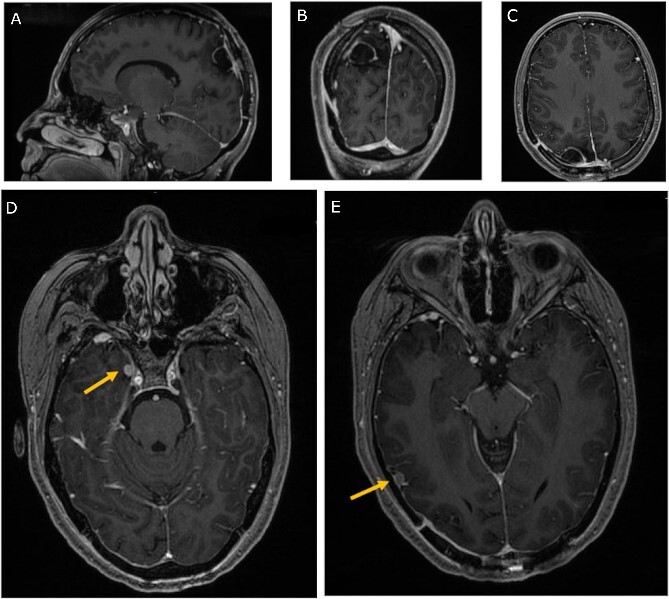
Three-month follow-up MRI; (A) sagittal, (B) coronal, and (C) axial post-contrast T1-weighted MRI scans show peripheral contrast enhancement at the parietal resection margin with areas of nodularity related to postoperative changes without recurrence; (D, E) axial post-contrast T1-weighted MRI shows stability of the medial and lateral temporal lobe lesions.

Chemotherapy and radiation therapy were not recommended due to the low-grade features on pathological examination. The patient was then discharged home at her pre-operative neurological baseline. The 3-month follow-up MRI showed no recurrence of the mass and stability of the right temporal lesions (Fig. 4).

## Discussion

First described by Limas and Tio [5], ultrastructural studies showed that intracranial melanocytomas are derived from leptomeningeal melanocytes which are most concentrated in the posterior fossa and upper cervical spinal cord, and thus most commonly arise in these locations. The supratentorial region is an extremely rare location for primary meningeal melanocytoma, with only a few similar cases being reported thus far in the frontal, temporal, and parietal lobes (Table 1).

**Table 1:** Literature review of seven cases of supratentorial meningeal melanocytoma.

**Reference**	**Age (years)/Sex**	**Location**	**MRI (T1)**	**MRI (T2)**	**Operation**
Rahimi-Movaghar and Karimi, 2003	17/M	Left parietal	NA	NA	Gross total
Munoz-Hidalgo *et al.*, 2014 [11]	15/M	Two lesions in right temporal	Lesion 1: Hyperintense Lesion 2: Hypointense	Lesion 1: Isointense Lesion 2: Hypointense	Gross total
Samadian *et al.*, 2015 [12]	19/M	Left temporal	Hyperintense	Isointense	Gross total + dural coagulation
Shin *et al.*, 2015 [13]	56/F	Left temporal	NA	Isointense	Gross total + dural excision
Pierecchi-Mart *et al.*, 2002 [3]	25/M	Left frontal	NA	NA	Gross total
Navas *et al.*, 2009 [14]	25/M	Right frontotemporal	Hyperintense	Hypointense	Gross total
Ken-Liang Kuo [15]	51/F	Right frontal	Hyperintense	Hypointense	Gross total + dural excision

NA = not applicable (no reported findings available)

It is also unusual for multiple primary meningeal melanocytomas to be found simultaneously in several discrete regions of the brain. While there have been previous cases of recurrent lesions arising after resection of the initial tumor, there have thus far been no reports of multiple primary melanocytomas co-occurring in more than one supratentorial location [6]. The presence of multiple lesions may often be interpreted as metastatic melanoma as opposed to a more benign primary meningeal melanocytoma. Although this feature is suggestive of metastasis, Terao *et al*. [7] reported other clinical distinctions between metastatic and primary melanocytic lesions in the central nervous system: metastatic melanomas typically develop in older individuals (>50 years) and are often found in the context of diffusely metastatic disease conferring a poorer prognosis. This case therefore highlights the importance of considering factors other than the multiplicity of lesions, such as patient demographics and previous history of cutaneous melanoma, when differentiating between primary and metastatic melanocytic disease.

Primary meningeal melanocytoma can also be difficult to differentiate from meningioma as they are both dural-based. Lin *et al*. [2] initially diagnosed a meningeal melanocytoma in the anterior cranial fossa as a meningioma, but later established the correct diagnosis through retrospective review of MR images. However, primary meningeal melanocytomas display characteristic patterns on imaging that may help distinguish these entities. Meningeal melanocytomas present with a high signal on T1- and FLAIR-weighted MRI, and a low signal on T2-weighted MRI [8] The imaging in the present case was consistent with these previous findings and thus lends further support to the idea that this tumor has distinct, predictable radiological characteristics which may narrow the differential diagnosis to guide histopathological confirmation.

The histopathological, immunohistochemical, and genetic characteristics of the tumor in this case are consistent with known features: the tumor consisted of heavily pigmented spindle/oval tumor cells staining positive for MelanA and HMB45 and exhibited a pathogenic GNAQ mutation [9]. The tumor was negative for a mutation in the BRAF gene, which is consistent with the lack of cutaneous melanomatous lesions in this patient. BRAF mutations are present in ~50% of melanomas [10] but typically not in melanocytomas, although this has been reported on those melanocytomas associated with the nevus of Ota in neurocutaneous syndromes [11].

Although benign, tumor recurrence and malignant transformation have been reported. Deng *et al*. [6] reported a case of a patient who had recurrence of meningeal melanocytoma 9 months after a gross total resection with intracranial spread. Resection of the recurrent lesion revealed malignant transformation to malignant melanoma. In our case, although the risk of malignant transformation was unknown prior to histological assessment, the indications for gross total resection of the tumor included relief of symptoms related to mass effect and obtaining tissue for pathological diagnosis. Alike to most of the previously described cases of primary meningeal melanocytoma, the patient presented herein had benign findings on pathological assessment and a stable clinical course at 4 months of follow-up.

## Conclusion

We describe the clinical characteristics, management strategy, and histopathological features of a rare case of a patient with multiple primary intracranial melanocytoma lesions. The case presented herein highlights the utility of specific imaging criteria such as diffusely increased T1 signal without enhancement in the initial diagnostic evaluation of intracranial melanocytoma.
